# KIF2A silencing inhibits the proliferation and migration of breast cancer cells and correlates with unfavorable prognosis in breast cancer

**DOI:** 10.1186/1471-2407-14-461

**Published:** 2014-06-21

**Authors:** Jianli Wang, Siqin Ma, Rong Ma, Xun Qu, Wenjun Liu, Cuixia Lv, Song Zhao, Yunyun Gong

**Affiliations:** 1 Department of Pathology and Pathophysiology, School of Medicine, Shandong University, 44# Wen Hua Xi Road, Jinan, Shandong, China; 2 School of Stomatology, Shandong University, Jinan, Shandong, China; 3 Department of Breast Surgery, Qilu Hospital, Shandong University, 107# Wen Hua Xi Road, Jinan, Shandong, China; 4 Institute of Basic Medical Science, Qilu Hospital, Shandong University, Jinan, Shandong, China; 5 Shandong Center for Disease Control and Prevention, Jinan, Shandong, China; 6 Institute for Global Food Security, School of Biological Sciences, Queen’s University of Belfast, Belfast, UK

**Keywords:** KIF2A, Breast cancer, Migration, Invasion, Survival, Marker

## Abstract

**Background:**

Kinesin family member 2a (KIF2A), a type of motor protein found in eukaryotic cells, is associated with development and progression of various human cancers. The role of KIF2A during breast cancer tumorigenesis and progression was studied.

**Methods:**

Immunohistochemical staining, real time RT-PCR and western blot were used to examine the expression of KIF2A in cancer tissues and adjacent normal tissues from breast cancer patients. Patients’ survival in relation to KIF2A expression was estimated using the Kaplan–Meier survival and multivariate analysis. Breast cancer cell line, MDA-MB-231 was used to study the proliferation, migration and invasion of cells following KIF2A-siRNA transfection.

**Results:**

The expression of KIF2A in cancer tissues was higher than that in normal adjacent tissues from the same patient (*P* < 0.05). KIF2A expression in cancer tissue with lymph node metastasis and HER2 positive cancer were higher than that in cancer tissue without (*P* < 0.05). A negative correlation was found between KIF2A expression levels in breast cancer and the survival time of breast cancer patients (*P* < 0.05). In addition, multivariate analysis indicated that KIF2A was an independent prognostic for outcome in breast cancer (OR: 16.55, 95% CI: 2.216-123.631, *P* = 0.006). The proliferation, migration and invasion of cancer cells *in vitro* were suppressed by KIF2A gene silencing (*P* < 0.05).

**Conclusions:**

KIF2A may play an important role in breast cancer progression and is potentially a novel predictive and prognostic marker for breast cancer.

## Background

Breast cancer is the leading cause of cancer-related death in Southeast Asian women, and is second only to gastric cancer in East Asian women [1]. Breast cancer incidence rates have been increasing annually, and in some countries, breast cancer is the most common cause of death among women [2,3].

In recent years, several members of the kinesin family of motor proteins have been identified in mammalian cells. In particular, it has been shown that the kinesin-13 class of proteins, which includes KIF2A, KIF2B, and KIF2C/MCAK, play an important role in mitosis [4,5]. These proteins modulate intracellular transport, cell division, and bipolar spindle assembly during spindle formation [5-7]. During cancer cell proliferation, the chromosome appears to be unstable as a result of continuous chromosomal missegregation during mitosis [8,9]. As a critical cytoskeleton, microtubules (MTs) are not only essential for mitotic activity of malignant cells but also for invading neighboring tissues and for the distant metastasis. The KIFs participate in spindle orientation and chromosomal movements during mitosis and cytoskeletal reorganization [10-13]. KIF2A has been shown to have microtubule depolymerizing activities *in vitro*[14,15]. Monopolar spindles in the cells could cause the gain or loss of chromosomes in daughter cells [16]. Any interference with this process can lead to chromosome missegregation, resulting in significant changes in the proliferation and migration of tumor cells. Among kinesin family members, the function of KIF2C/MCAK in gastric, colorectal and breast cancers has been studied. However, the role of KIF2A in breast cancer remains unknown. The goal of this study was to explore the function of KIF2A in human breast cancer, and to determine its effects on the proliferation and invasion of breast cancer cells.

## Methods

### Breast cancer patient study

#### ***Tissue sample collection and survival data collection***

One hundred and twenty female patients with primary breast cancer of the infiltrating ductal carcinoma type according to the diagnostic criteria of WHO Classification of Tumors of the breast (The Fourth Edition, 2012), participated in the study immediately after surgery at Qilu Hospital of Shandong University, and were followed up until the study ended. Patients did not receive chemotherapy or radiotherapy before surgery. The median age of the patient at surgery was 46 years (range 20–71). By the time the study ended, the 120 women had been followed up for 45–114 months (median 55 months). Patient age, tumor size, histological grade, and nodal metastasis status were recorded. Specimens from cancer tissue and adjacent normal tissue were obtained during the surgery and were immediately dipped in 10% formalin for immunohistochemistry analysis. Twelve patients had both cancer and adjacent tissue sufficiently large so that a portion of the tissue was immediately frozen in liquid nitrogen and then stored at -80°C for RNA/protein analysis. Written informed consent was obtained from all patients. Qilu Hospital of Shandong University Ethics Committee approval was obtained for the study.

#### ***KIF2A gene expression and protein in breast cancer tissues and adjacent tissues***

Total RNA and protein were extracted from the 12 pairs of breast cancer tissues and adjacent normal tissue. Briefly, total RNA was isolated using TRIzol reagent (Invitrogen, Carlsbad, CA, USA) and reverse transcriptase reactions were carried out with 1μg RNA, M-MuLV reverse transcriptase SuperscriptII (Invitrogen, USA) in a volume of 20 μL. For real-time RT-PCR, the LightCycler 2.0 instrument (Roche Applied Science, Mannheim, Germany) and the SYBR Premix Ex Taq™II (TaKaRa Bio Group, Japan) were used according to the manufacturer’s instructions. The KIF2A primer *5’-GCCTTTGATGACTCAGCTCC-3’* (sense), and *5’-TTCCTGAAAAGTCACCACCC-3’* (antisense) sequences were obtained from Shanghai Ji kai Biological Engineering Technology & Services Co, China. The specific primer sets for β-actin were as follows: sense: *5’-CATGTACGTTGCTATCCAGGC-3’*, and antisense: *5’-CTCCTTAATGTCACGCACGAT-3’*. The reactions were incubated in a 96-well optical plate at 95°C for 5 min, followed by 40 cycles of 95°C for 30 s, 60°C for 45 s and 72°C for 30 s. Experiments were performed in duplicate for each sample and C_t_ data of each sample was determined using default threshold settings. The relative quantification was given by the C_t_ values, determined by triplicate reactions for all of the samples for both KIF2A and β-actin. The ΔC_t_ was determined by subtracting the β-actin C_t_ from the target gene C_t_. The relative expression level of KIF2A mRNA was determined by 2^-ΔCt^. The fold change of KIF2A expression calculation = the mean of relative KIF2A gene expression in cancers/the mean of relative KIF2A gene expression in adjacent normal tissues.

The protein extracted from the above paired tissues was measured using the Western Blotting method with an anti-human KIF2A rabbit polyclonal antibody (dilution 1:10,000, Abcam; Cambridge, UK) as the primary antibody and a peroxidase-conjugated anti-rabbit antibody (1:2000, Santa-Cruz CA) as secondary antibody. Extracted protein (50μg) was loaded onto the gels. The protein intensity was detected by enhanced chemiluminescence (ECL) and quantified by Bio-Rad ChemiDoc™ Image Lab Software. Glyceraldehyde-3-phosphate dehydrogenase (GAPDH, dilution 1:3,000, incubated overnight at 4°C, Proteintech Group, INC. Chinese subsidiary) expression was used as a loading control. The protein signal intensity was quantified by JD801 Analysis Software (Jie Da Science and Technology Development Co., Ltd. Jiangsu province, China) and replicated three times.

#### ***Immunohistochemistry (IHC) and fluorescence in situ hybridization (FISH) analysis in cancer tissues***

KIF2A was determined by an IHC method [17] in cancer tissues and adjacent tissues from the 120 samples. The adjacent normal tissue was sampled at least 1 cm distance from the tumor to ensure no tumor tissue is included in the slide. Estrogen receptor (ER), progesterone receptor (PR) and human epidermal growth factor receptor 2 (HER2) status were examined by the IHC method in cancer tissues, and if necessary, fluorescence in situ hybridization (FISH) of HER2 in cancer tissues. FISH was performed using the HER2 DNA dual-colored Probe Kit (Abbott Molecular Inc., Des Plaines, IL,USA). Anti-human KIF2A rabbit polyclonal antibody (dilution 1:500, Abcam) and monoclonal antibodies directed against ER/PR (Dako) were used for these assays. Omission of primary antibody was used as a negative control. The slides were scored by a pathologist who was blind to the patient outcome, using the Harvey scoring system to evaluate KIF2A IHC staining [18]. A score was assigned to represent the estimated proportion of positively stained tumor cells: none = 0; 1⁄100 = 1; 1⁄100 to 1/10 = 2; 1⁄10 to 1⁄3 = 3; 1⁄3 to 2⁄3 = 4; and 2⁄3 = 5. An intensity score was assigned based on the average intensity of positive tumor cells: 0 = none; 1 = weak, 2 = intermediate; and 3 = strong (Additional file 1: Figure S1). The proportion and intensity scores were added to obtain a total score, which ranged from 0 to 8. The expression of KIF2A in the majority of adjacent tissue was scored as 4–6. All breast cancer tissue scores ranged from 6–8, hence, ≤6 was considered low, and >6 was considered high expression.

HER2 protein levels on the tumor cell surface were measured by IHC, initially, using a HER2-specific antibody. HER2 immunohistochemistry staining was scored as 0, 1+, 2+, or 3+ based on the percentage of stained malignant cells, and the degree of membrane staining present in these malignant cells. A tumor with an IHC score of 0 or 1+ was considered HER2 negative. Tissues with an IHC score of 2+ were subject to further FISH testing to determine HER2 status. A tumor with an IHC score of 3+ or a FISH ratio greater than 2.2 was considered to be HER2 positive [19].

### Experimental study

#### ***Cell culture***

Human breast cancer cell lines MCF-7, MDA-MB-231, T47D and MDA-MB-468 were purchased from the Tumor Cell Library of the Chinese Academy of Medical Sciences (Beijing, China) and were cultured in RPMI-1640 (MCF-7) and DMEM (MDA-MB-231,T47D, MDA-MB-468) containing 10% fetal calf serum (Gibco, Invitrogen, Carlsbad, CA, USA) at 37°C in 5% CO_2_.

#### ***KIF2A expression in breast cancer cell lines***

Total RNA from MCF-7, MDA-MB-231, T47D and MDA-MB-468 were extracted using Trizol reagent. 1 μL cDNA from the reverse transcription (see above) was applied as template. PCR cycling conditions began with an initial cDNA denaturation step at 94°C for 4 min followed by 30 cycles with denaturation at 94°C for 30 s, primer annealing at the 55°C for 30 s, extension at 72°C for 45 s and followed by a final extension step at 72°C for 5 min. The amplified PCR products were analyzed by separation in a 2% agarose gel at 80 V for 30 min. The intensity of RT-PCR band was determined using JD801 Analysis Software. The comparison of KIF2A gene expression among these cell lines suggested that KIF2A expression was high in MDA-MB-231; therefore, this cell line was chosen for the following experiments.

#### ***KIF2A Gene silencing by siRNA***

KIF2A gene silencing was studied to exclude the possibility of an off-target effect by KIF2A-siRNA. The two target sequences of the synthetic oligonucleotides for siRNA *5’-GGCAAAGAGAUUGACCUGG-3’*(siRNA-1#) and *5’-CCCUCCUUCAAGAGAUAAUTT-3’* (siRNA-2#) (Ambion, Austin, TX, USA) had similar effects (Additional file 2: Figure S2), hence the first sequence (50 nM) was employed in our experiments. Nonsense-siRNA (Dharmacon, Lafayette, Colorado, USA) and mock treatment were used as negative controls. MDA-MB-231 cells, upon reaching 2 × 10^5^ cells per well in six-well plates, were transfected with KIF2A–siRNA and nonsense-siRNA using Lipofectamine 2000 (Invitrogen, Carlsbad, California, USA) in Opti-MEM (Invitrogen). Cells in the mock group were only treated with medium. After 48 hours of treatment, the cells were harvested for gene expression (RT-PCR method see above) and protein expression analysis (Western Blotting method see cancer tissue protein analysis).

#### ***Cell proliferation study***

Following KIF2A silencing with siRNA, cell proliferation was measured using the 3-(4,5-dimethylthiazol-2-yl)-2,5-diphenyltetrazolium bromide (MTT) assay (Amresco, Solon, OH, USA). After incubation for 0, 24, 48, and 72 hours, optical densities were read at wavelength 570 nm.

### Migratory and invasive ability assays

#### ***Cell migration assay***

The migration of MDA-MB-231 cells following KIF2A gene silencing, were evaluated in 24-well Boyden chambers containing polycarbonate filters with a pore size of 8.0-μm (BD Biosciences, San Jose, California, USA). The different cell groups, including mock, nonsense-siRNA and KIF2A–siRNA, were seeded in the top chamber of the transwell with 2 × 10^5^ cells per chamber, and the lower chamber was filled with 600 μl DMEM with 10% fetal calf serum as a chemoattractant. Cells were allowed to migrate for 16 hours. At the end of the incubation period, the cells in the upper chamber were removed by wiping gently with a cotton swab. Cells that had migrated to the bottom side of the membrane were fixed in methanol for 10 minutes and stained with eosin. The migrated cells at the lower surface were counted under a light microscope at a 200× magnification. Cells were counted blind in 6 different random fields. All experiments were done in triplicate.

#### ***Cell matrigel invasion assay***

The invasive ability of MDA-MB-231 cells, following gene silencing, was determined by the same Boyden transwell chambers as used for the migration assay. The filters were coated with 40 μl of Matrigel, and incubated in a humidified incubator at 37°C to allow polymerization. The lower compartment was filled with DMEM supplemented with 10% FBS. Different MDA-MB-231 cell groups, mock, nonsense-siRNA and KIF2A–siRNA were in 0.2 ml serum free DMEM and seeded on top of the collagen in the upper compartment of the chamber (2 × 10^5^ cells/chamber). After incubation at 37°C for 24 h, the cells on the upper surface of the filter were removed by wiping. Cells that traversed the filter were fixed in methanol, stained with 0.5% eosin and counted (see migration).

#### ***Statistical analysis***

All statistical analyses were performed using the SPSS17.0 software package for Windows. All values are presented as mean ± standard deviation (SD) and the P-values are from two-sided tests. Pairwise comparison was applied to the difference between cancer tissues and adjacent tissues. Student t-test or ANOVA was used for comparison of two or more groups in continuous variables. Kaplan Meier survival analysis was used to obtain the survival curves. Multivariate Cox analysis was used to examine the relationship between survival time and pathological characteristics. A P-value less than 0.05 was considered statistically significant.

## Results

### Breast cancer patient study

#### ***KIF2A mRNA and protein expression***

**Figure 1:**
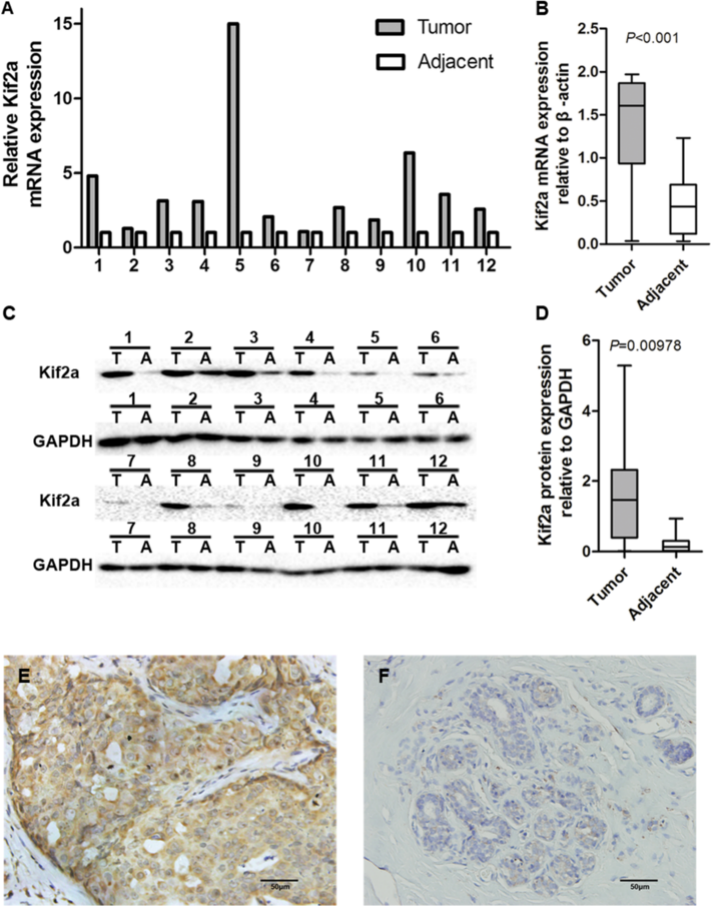
**KIF2A protein and mRNA expression in breast cancer specimens.** The KIF2A mRNA levels were significantly upregulated in breast cancer tissues than in adjacent tissues (**A**, **B**, *P* < 0.001). KIF2A protein expression was upregulated in breast cancer as determined by Western Blot (**C**, **D**, *P* < 0.001). Stronger KIF2A expression was observed in cancer cells **(E)** than in adjacent normal breast tissue **(F)** using immunohistochemistry method. IHC 400×. T = tumor, A = adjacent (normal tissue).

We detected the KIF2A mRNA and protein levels in 12 paired primary breast cancer tissues and the corresponding adjacent normal tissues using real time RT-PCR and western blotting, respectively. KIF2A mRNA was up-regulated in primary breast cancer tissue, by 2.95 fold the expression in adjacent normal breast tissues (Figure 1A, B, *P <* 0.001). Consistently, the western blotting analysis showed that the KIF2A protein levels in primary breast cancer tissues were markedly higher than those in the adjacent normal breast tissues (Figure 1C, D, *P <* 0.001). These results suggested that KIF2A is overexpressed in breast cancer tissues.

#### ***KIF2A IHC results***

IHC was performed to determine KIF2A protein expression in 120 primary breast cancer tissues. The KIF2A expression level in primary cancer tissues (Figure 1E) was higher than that in the adjacent epithelial tissue (Figure 1F), with mean scores at 7.1 ± 0.7 vs. 6.3 ± 1.0 (*P <* 0.05). The IHC results showed that 80 of 120 (67%) cases of breast cancer overexpressed KIF2A compared with the paired normal adjacent tissues.

#### ***The relationship between KIF2A expression and clinical pathological factors***

The KIF2A expression in relation to the key clinico-pathologic factors of breast cancer, such as age, tumor size, tumor histological grade, clinical stage, ER, PR and HER2 status, was examined in the 120 patients using univariate and multivariate analysis, (see Table 1). HER2 positive patients had significantly higher KIF2A mRNA expression levels than those that were HER2 negative (*P* = 0.019). There were no significant correlations between KIF2A expression and other clinicopathological factors. Patients with positive lymph node metastasis had higher KIF2A expression levels than those who were negative (7.31 ± 0.66 vs 6.69 ± 0.72, *P* < 0.001). The overexpression of KIF2A in adjacent tissues was more frequently observed in patients with lymph node metastasis than those without lymph node metastasis (6.52 ± 1.01 vs 5.91 ± 0.78, *P* < 0.001). See Additional file 3: Figure S3.

**Table 1:** KIF2A expression in 120 cancer tissues and pathological characteristics

**Variable**	**n**	**KIF2A expression (scores)**	**Multiple regression analysis**
		**Mean ± SD**	**P-value**
Age			
<50	75	7.09 ± 0.76	0.533
≥50	45	7.00 ± 0.74	
Tumor size(cm)			
≤2	44	6.89 ± 0.62	0.571
>2	72	7.18 ± 0.79	
>5	4	6.75 ± 0.96	
Tumor grade			
I	30	7.03 ± 0.81	0.071
II	73	7.10 ± 0.71	
III	17	6.94 ± 0.83	
Lymph metastasis			
Positive	72	7.31 ± 0.66	<0.001^**^
Negative	48	6.69 ± 0.72	
ER			
Positive	81	7.02 ± 0.74	0.802
Negative	39	7.13 ± 0.77	
PR			
Positive	68	7.00 ± 0.73	0.749
Negative	52	7.13 ± 0.77	
HER2			
Positive	30	7.33 ± 0.76	0.047^*^
Negative	90	6.97 ± 0.73	
Total	120		

^*^difference between the two groups is statistically significant, *P* = 0.047.

^**^difference between the two groups is statistically significant, *P* < 0.001.

#### ***Association of KIF2A expression with survival***

**Figure 2:**
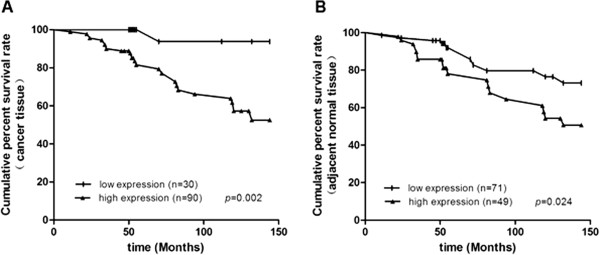
**Kaplan-Meier analysis of cancer-specific survival.** KIF2A protein levels in primary cancer tissues **(A)** and paired adjacent normal tissue **(B)** showed prognostic roles in overall survival. Patients with high KIF2A expression in cancer and adjacent tissue had a significantly poorer prognosis than those with low KIF2A expression (*P*_cancer_ = 0.002, *P*_adjacent tissue_ = 0.024).

KIF2A expression group based on whether her IHC score was above 6 or not. The survival time was significantly lower in the KIF2A high expressing patients overall (*P* < 0.05). Patients with high KIF2A expression in breast cancer tissues (Figure 2A) or adjacent normal tissues (Figure 2B) were more likely to have significantly poor survival (*P* = 0.002 when high KIF2A in cancer tissues, and *P* = 0.024 when in adjacent tissues).

#### ***Univariate and multivariate survival analyses***

We evaluated the KIF2A expression and other clinicopathologic factors on prognosis of breast cancer by univariate analyses. Results indicated that histological grade (OR: 2.347, 95% CI: 1.351-4.076, *P* = 0.002), lymph node status (OR: 3.273, 95% CI: 1.328-8.066, *P* = 0.010), ER (OR: 0.360, 95% CI: 0 .170-0.758, *P* = 0.007) and KIF2A (OR: 11.699, 95% CI: 1.590-86.058, *P* = 0.016) as significant predictors of cancer-specific survival. Patient age, tumor size, PR and HER2 did not significantly affect cancer-specific survival (Table 2). Furthermore, multivariate statistical analysis revealed that high expression of KIF2A and histology grade were closely related to overall survival. The high expression of KIF2A (OR: 16.55, 95% CI: 2.216-123.631, Table 2) in breast cancer tissue was an independent prognostic factor for breast cancer in addition to the patient’s tumor histological grade (OR: 3.108, 95% CI: 1.712-5.643, Table 2).

**Table 2:** Cox regression analyses for survival time

	**Univariate**		**Multivariate**	
**Variable**	**OR (95% CI)**	**P-value**	**OR (95% CI)**	**P-value**
Age >50	1.289(0.620-2.680)	0.497		
Tumorsize(2/>2/>5)	1.594(0.705-3.601)	0.262		
Grade(I/II/III)	2.347(1.351-4.076)	0.002	3.108(1.712-5.643)	<0.001
Lymph metastasis	3.273(1.328-8.066)	0.010		
ER negative	0.360(0 .170-0.758)	0.007		
PR negative	0.561(0.270-1.166)	0.122		
HER2 negative	1.923(0.918-4.030)	0.083		
KIF2A highexp*	11.699(1.590-86.058)	0.016	16.550 (2.216-123.631)	0.006

*KIF2A high expression is score > 6.

### Experimental studies

**Figure 3:**
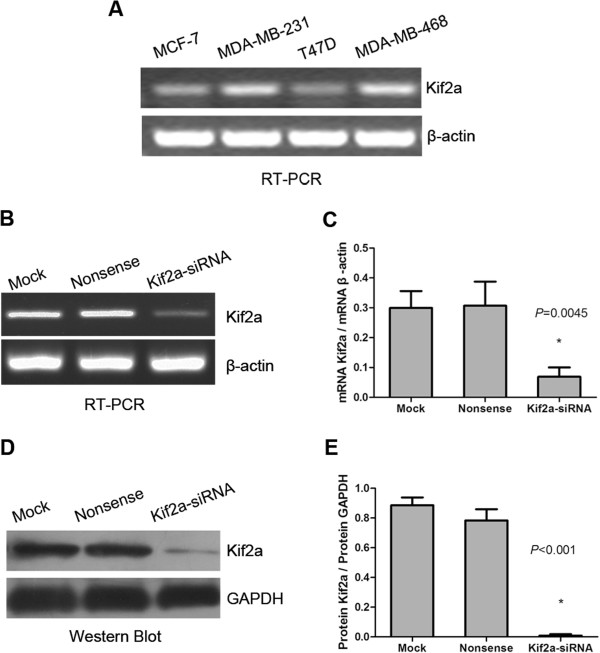
**KIF2A gene silencing with KIF2A–siRNA.** The cell lines MCF-7, MDA-MB-231, T47D and MDA-MB-468 were screened by RT-PCR for expression of KIF2A, with MDA-MB-231 showing the strongest expression level **(A)**. MDA-MB-231 cells were transfected with mock, nonsense-siRNA, and KIF2A–siRNA. The expression of KIF2A mRNA **(B)** and protein **(D)** in the group transfected with KIF2A–siRNA decreased significantly compared to the two control groups (*P*_mRNA_ = 0.0045, *P*_protein_ < 0.001). β-Actin and GAPDH were used as controls. Data were expressed as the relative ratio of KIF2A mRNA and protein to that of β-actin and GAPDH **(C, E)**. All experiments were done in triplicate.

RT-PCR and western blot analysis of KIF2A expression in MCF-7, MDA-MB-231, MDA-MB-468 and T47D breast cancer cell lines are shown in Figure 3A. Because it had the highest expression of KIF2A, the MDA-MB-231 breast cancer cell line was used for the *in vitro* experiments in this study.

### Effect of gene silencing using siRNA

**Figure 4:**
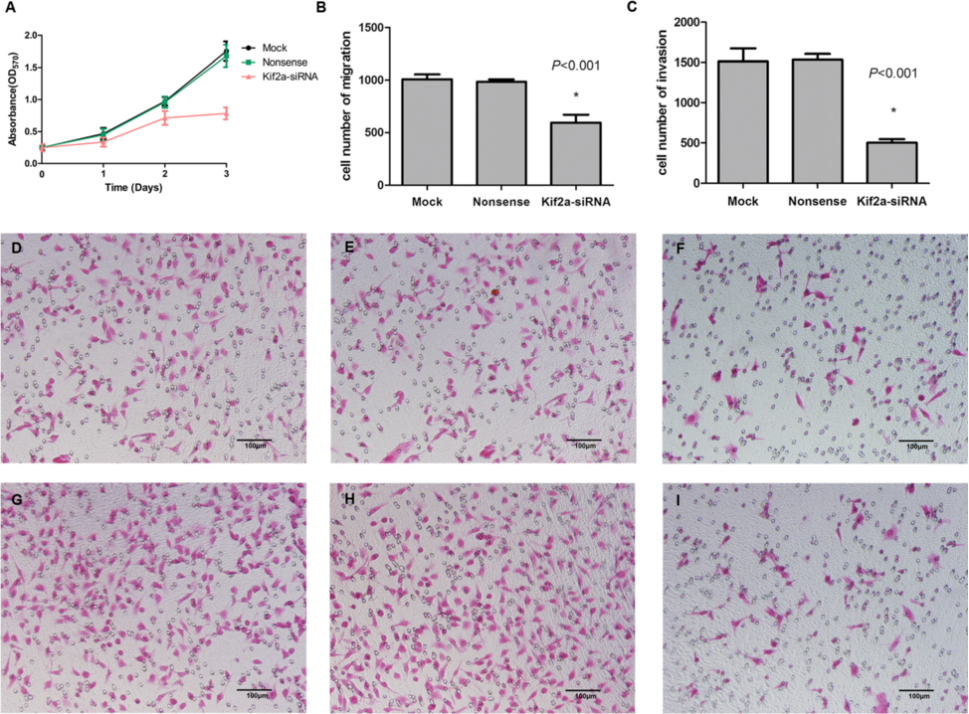
**The effects of KIF2A silencing on cell proliferation, migration and invasion.** The cell proliferation rate at 12, 24 and 36 hours, respectively, was inhibited in KIF2A-siRNA transfected cells **(A)**. The numbers of migrating **(B)** and invasive **(C)** cells in the mock and nonsense-siRNA groups were significantly higher than those in the KIF2A-siRNA transfected group (*P* < 0.001). The data are representative of three experiments. Microscope images (with EOSIN staining, 200×) showing the mock, the nonsense-siRNA and the KIF2A-siRNA transfected cells in the migratory assay **(D-F)**, and in the invasive assay **(G-I)**.

KIF2A-siRNA was transfected into MDA-MB-231 cells, and after 48 hours,KIF2A mRNA and protein levels in siRNA-transfected cells were significantly decreased compared with mock group, see the mRNA (Figure 3B, C) and protein levels (Figure 3D, E). These results indicated that KIF2A–siRNA had targeted gene-silencing effects on KIF2A.The effect of KIF2A knockdown on MDA-MB-231 cell proliferation is shown in Figure 4A, where it can be seen that proliferation of the knockdown cells was lower than the mock and nonsense treated cells at 24, 48, and 72 hours in a time-dependent trend, whereas the control cells continued to proliferate. There was no significant difference between mock cells and nonsense-transfected cells.

#### ***Migratory and invasive ability***

Silencing KIF2A resulted in a decrease in cell migration and invasion compared to the control groups. Migratory ability was decreased significantly in KIF2A-siRNA treated cells compared to the mock and nonsense-siRNA groups (*P* < 0.001) (Figure 4B, D-F). The number of cells that invaded the matrigel membrane was significantly lowered in KIF2A-siRNA treated cells compared with the mock and nonsense-siRNA groups (*P* < 0.001) (Figure 4C, G-I).

## Discussion

In this study, we found that the mitotic centromere-associated Kinesin-13 protein KIF2A mRNA and protein levels in breast cancer tissue were highly expressed compared to adjacent normal tissue. The increase in KIF2A expression was associated with a decrease in patient survival time indicating that KIF2A is a potential novel prognosis biomarker for breast cancer. We also demonstrated that patients with lymph node metastasis had high frequency of KIF2A overexpression.

As the key components of the cytoskeleton, MTs play important roles in mitosis, cell migration, trafficking and cell signaling [20]. Kinesin is one family of motor protein associated with MT transport. The Kinesin-13 proteins (KIF2A, KIF2B, and KIF2C/MCAK) are MT depolymerases that depolymerize the ends of MTs [21,22] and have important functions in mitosis, including the catalysis of microtubule disassembly, which is important for normal chromosome movement [23]. KIF2A was found to specifically localize in centrosomes during the process of cellular mitosis, and possesses microtubule-depolymerizing activity for spindle bipolarity [14,16]. The overexpression of these proteins causes a moderate increase in the frequency of multipolar and monopolar spindles [24,25], which may further contribute to the gain or loss of chromosomes in daughter cells. In a study by Ganem et al. [16]*,* the cell cycle was inhibited in cells with RNAi-induced knockdown of KIF2A, and these cells formed monopolar spindles instead of bipolar spindles, leading to chromosome mis-segregation in cells.

Human osteosarcoma (U2OS) cells transfected with non-sense siRNA typically had bipolar spindles but 90% of cells transfected with KIF2A-specific siRNA had monopolar spindles [16]. KIF2B showed a similar effect as KIF2A in the study conducted by [5] and KIF2C is overexpressed in gastric cancer cells [26], which may enhance cellular proliferation by increasing the rate of cancer cell mitosis. To demonstrate the function of KIF2C in cancer cell proliferation, Shimo et al. knocked down endogenous KIF2C/MACK in breast cancer cells [7], which led to a significant decrease in cell proliferation. These results illustrated the contribution of KIF2C to the aggressive behavior of KIF2C-overexpressing breast tumors. The formation of monopolar spindles and inhibition of cellular proliferation were also observed in human cancer cells treated with anti-KIF2A siRNA or Xenopus eggs with a KIF2A antibody [27,28]. We reported that the migratory ability of KIF2A–siRNA transfected oral cancer cells decreased significantly compared to the control group [29]. In the current study using MDA-MB-231 breast cancer cell, we found that upon transfection with KIF2A-siRNA, cell proliferation was inhibited, as was cell migration and invasion. Cells transfected with KIF2A-siRNA showed up to 55% reduction in cell growth, the migration decreased by 41%, and the invasion decreased by 66%. These findings suggest that the overexpression of KIF2A in breast cancer tissues may alter key features of the cells leading to uncontrolled proliferation, migration and invasion. KIF2A is likely to be an important growth factor and might be associated with the malignant phenotype of breast cancer cells. The fact that patients with lymph node metastasis had KIF2A overexpression in our study suggests that KIF2A overexpression correlates with an aggressive phenotype (Figure 2, Table 1). The follow-up results suggested that patients with KIF2A-overexpression had a poorer survival than patients without KIF2A-overexpressing.

Our current study demonstrated a relationship between HER2 and KIF2A expression in cancer cells (P < 0.05), which needs further investigation. HER2-positive breast cancers represent a unique subtype that are often associated with poorly differentiated, high-grade tumors, and have a poorer prognosis than HER2-negative type [17]. Histological grading provides important prognostic information, and patients with low grade tumors have a significantly better survival time than those with high grade tumors.

## Conclusions

In summary, metastasis. Patients with higher KIF2A expression have poorer survival. Cells with silenced KIF2A gene *in vitro* showed reduced cell proliferation, migration and invasion function, in agreement with the findings *in vivo*. The study provides clear evidence for the significant role of KIF2A in breast cancer development, using both patient data and a cell line model *in vitro.* The results indicate that KIF2A could potentially be an important novel prognostic marker for breast cancer.

## Competing interests

The authors declare that they have no competing interests.

## Authors’ contributions

WJL and MSQ were responsible for implementing cell culture. LWJ and ZS were responsible for IHC and following-up. WJL and LCX were responsible for data analyses. QX and GYY were responsible for experimental design. WJL and MR were responsible for the overall experimental design, data analysis, and implementation of the project. All authors read and approved the final manuscript.

## Pre-publication history

The pre-publication history for this paper can be accessed here:

http://www.biomedcentral.com/1471-2407/14/461/prepub

## Supplementary Material

Additional file 1: Figure S1 Immunohistochemical analysis of KIF2A in breast cancer tissues showing different intensity grade, (A) Weak, (B) moderate and (C) strong. IHC 400×. Click here for file

Additional file 2: Figure S2 KIF2A gene expression in MDA-MB-231 cells: KIF2A-siRNA-1# and siRNA-2# sequence have samilar effects on gene silenceing, detected by RT-PCR method. Click here for file

Additional file 3: Figure S3 Comparison of KIF2A IHC scores in breast cancer and adjacent tissue. The KIF2A levels were significantly upregulated in breast cancer tissues than in adjacent tissues (*P <* 0.001). All breast cancer tissue scores ≤6 was considered low, and >6 was considered high expression. Click here for file
