# Supplementary figures and images for: KIF2A silencing inhibits the proliferation and migration of breast cancer cells and correlates with unfavorable prognosis in breast cancer
Source: BMC Cancer. 2014 Jun 21;14:461. doi: 10.1186/1471-2407-14-461 (PMC4076253; doi:10.1186/1471-2407-14-461)

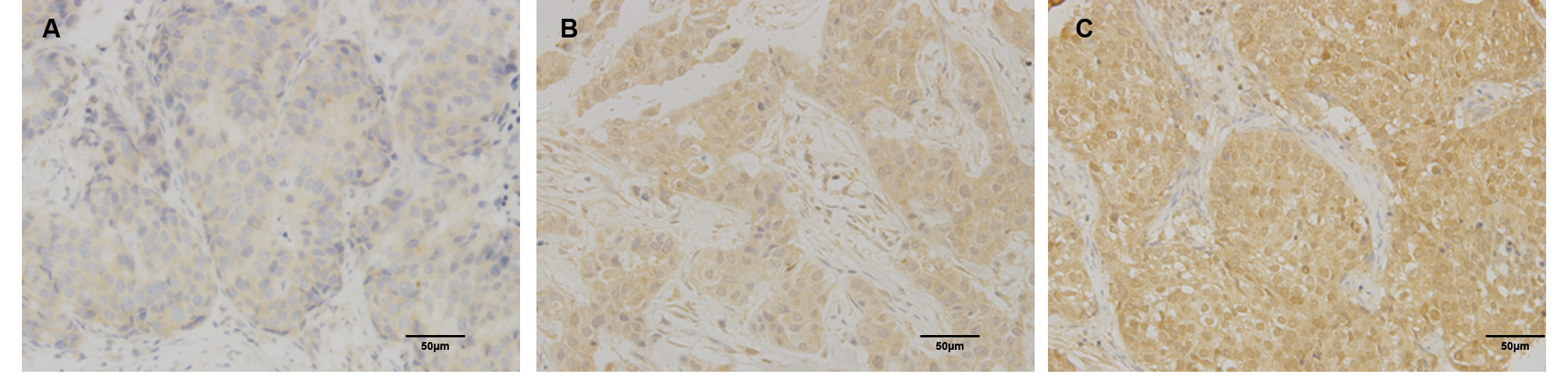

Supplement: Additional file 1: Figure S1 — Immunohistochemical analysis of KIF2A in breast cancer tissues showing different intensity grade, (A) Weak, (B) moderate and (C) strong. IHC 400×. [file 1471-2407-14-461-S1.tiff]

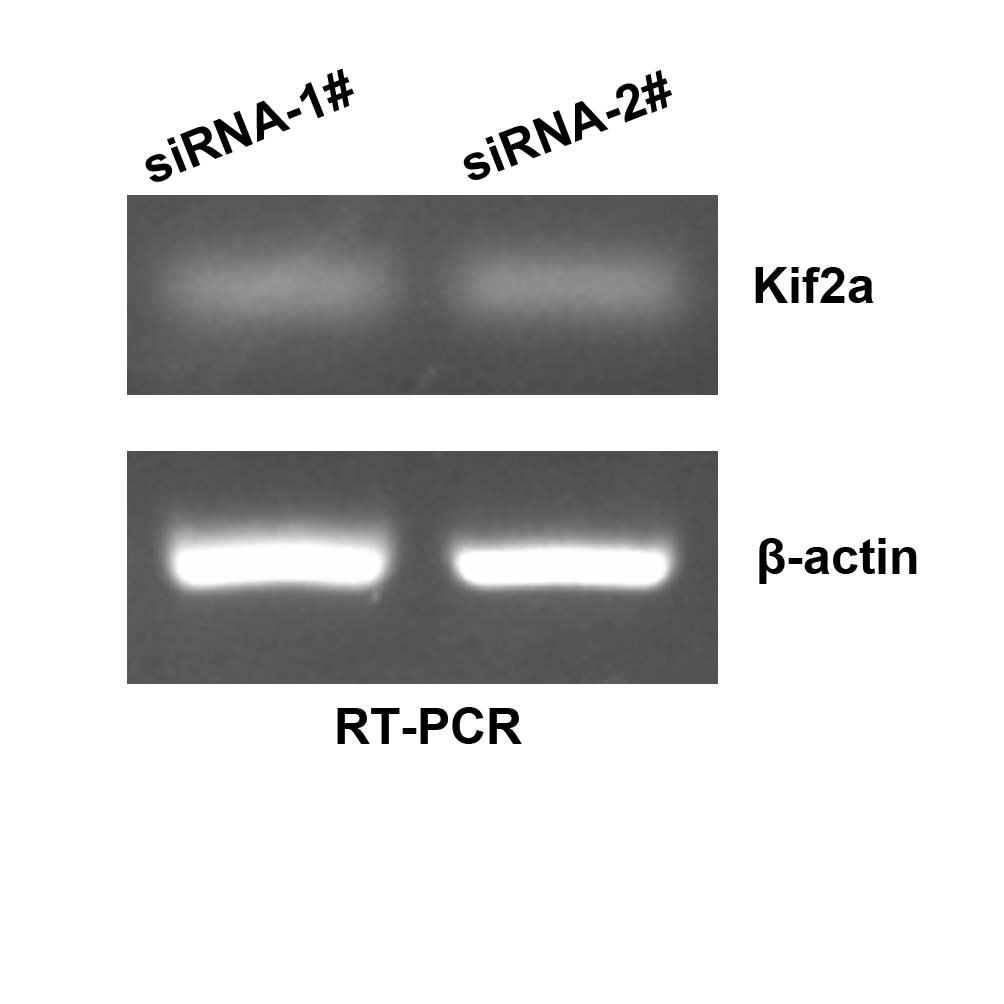

Supplement: Additional file 2: Figure S2 — KIF2A gene expression in MDA-MB-231 cells: KIF2A-siRNA-1# and siRNA-2# sequence have samilar effects on gene silenceing, detected by RT-PCR method. [file 1471-2407-14-461-S2.tiff]

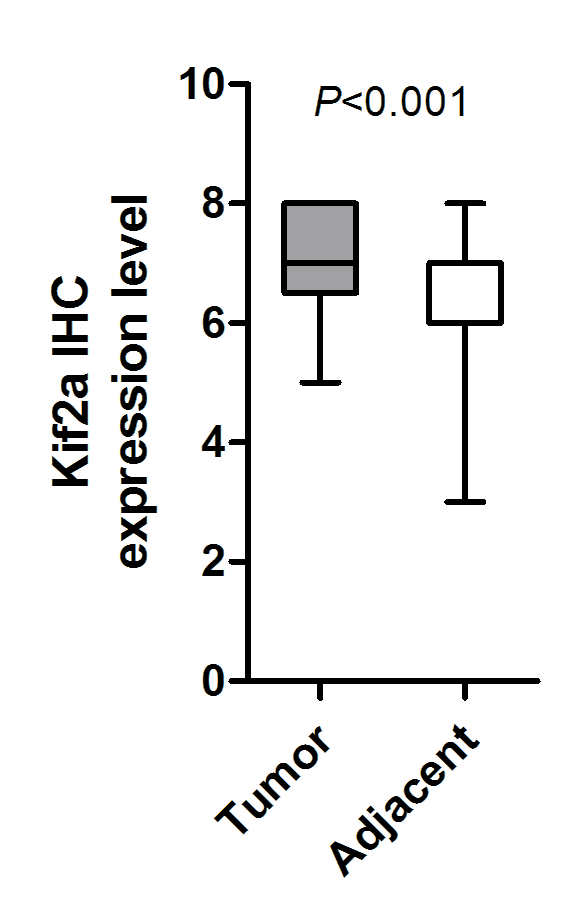

Supplement: Additional file 3: Figure S3 — Comparison of KIF2A IHC scores in breast cancer and adjacent tissue. The KIF2A levels were significantly upregulated in breast cancer tissues than in adjacent tissues (P < 0.001). All breast cancer tissue scores ≤6 was considered low, and >6 was considered high expression. [file 1471-2407-14-461-S3.tiff]
